# SrfB, a member of the Serum Response Factor family of transcription factors, regulates starvation response and early development in *Dictyostelium*

**DOI:** 10.1016/j.ydbio.2008.01.026

**Published:** 2008-04-15

**Authors:** María Galardi-Castilla, Barbara Pergolizzi, Gareth Bloomfield, Jason Skelton, Al Ivens, Robert R. Kay, Salvatore Bozzaro, Leandro Sastre

**Affiliations:** a Instituto de Investigaciones Biomédicas CSIC/UAM. Arturo Duperier, 4. 28029 Madrid, Spain; b Dip. Scienze Cliniche e Biologiche, Università di Torino, Ospedale S. Luigi, Orbassano, Italy; c MRC Laboratory of Molecular Biology, Hills Road, Cambridge, CB2 2QH, UK; d Wellcome Trust Sanger Institute, Cambridge, Cambridgeshire, CB10 1SA, UK

**Keywords:** *Dictyostelium*, SRF, Serum Response Factor, Transcription, Cytoskeleton, Actin, Differentiation, Development, cAMP, Aggregation

## Abstract

The Serum Response Factor (SRF) is an important regulator of cell proliferation and differentiation. *Dictyostelium discoideum srfB* gene codes for an SRF homologue and is expressed in vegetative cells and during development under the control of three alternative promoters, which show different cell-type specific patterns of expression. The two more proximal promoters directed gene transcription in prestalk AB, stalk and lower-cup cells. The generation of a strain where the *srfB* gene has been interrupted (*srfB*^*−*^) has shown that this gene is required for regulation of actin–cytoskeleton-related functions, such as cytokinesis and macropinocytosis. The mutant failed to develop well in suspension, but could be rescued by cAMP pulsing, suggesting a defect in cAMP signaling. *srfB*^*−*^ cells showed impaired chemotaxis to cAMP and defective lateral pseudopodium inhibition. Nevertheless, *srfB*^*−*^ cells aggregated on agar plates and nitrocellulose filters 2 h earlier than wild type cells, and completed development, showing an increased tendency to form slug structures. Analysis of wild type and *srfB*^*−*^ strains detected significant differences in the regulation of gene expression upon starvation. Genes coding for lysosomal and ribosomal proteins, developmentally-regulated genes, and some genes coding for proteins involved in cytoskeleton regulation were deregulated during the first stages of development.

## Introduction

The transcription factor Serum Response Factor (SRF) plays an important role in the regulation of differentiation and proliferation in mammalian cells. It was initially identified because of its role in the regulation of immediate early genes in response to growth factors (Treisman, 1987). Shortly afterwards SRF was found to regulate the expression of muscle-specific genes (Boxer et al., 1989). SRF binds DNA through the highly conserved DNA-binding and dimerization MADS-box domain (MCM1, ArgR1, in yeast, Agamous, Deficiens in plants and SRF in animals) (Treisman, 1995), which is conserved in plants, fungi, animals and amoebae (Alvarez-Buylla et al., 2000; Escalante and Sastre, 1998).

A large number of studies on SRF-dependent gene expression (Zang et al., 2005) and on SRF-binding-site containing promoters have allowed the identification of about 160 genes whose expression is regulated by SRF in mammals (Sun et al., 2007). Besides immediate early genes, SRF-dependent genes include many genes coding for components of the actin cytoskeleton and the muscular contractile apparatus (Miano et al., 2007). SRF regulates the expression of so many different genes through interaction with several signal-regulated and tissue-specific regulatory cofactors (Posern and Treisman, 2006).

Mice in which the SRF gene has been knocked down present embryonic lethality at gastrulation (Arsenian et al., 1998). Cells derived from mutant embryos show defects in cell adhesion, migration and cytoskeleton organization (Schratt et al., 2002). Tissue-specific SRF knockdowns have shown that SRF is required for terminal differentiation of skeletal-, cardiac-, and smooth-muscle cells and for the correct organization of the sarcomere (Miano et al., 2004; Li et al., 2005). SRF is also required for migration of neuronal cells (Alberti et al., 2005). These data, in conjunction with other studies in *Drosophila* and *Dictyostelium discoideum* support the proposal of considering SRF as a master regulator of the actin cytoskeleton and contractile apparatus (Miano et al., 2007).

The social amoeba *D. discoideum* belongs to the group of the Eumycetozoa, that diverged from the common branch of animals and fungi shortly after the separation of plants (Eichinger et al., 2005), These organisms grow in forest soils as amoebae but execute a complex multicellular developmental program upon starvation (Maeda, 2005). Cells aggregate by chemotaxis towards cAMP to form little mounds of up to 100,000 cells and undergo a cell-differentiation and morphogenetic program to give rise to a fruiting body consisting of a sorus supported on a cellular stalk. Formation of the fruiting body requires many processes shared with vertebrate embryogenesis, including cell migration, adhesion, inter-cellular signaling and coordinated cell differentiation (Chisholm and Firtel, 2004; Jin and Hereld, 2006).

A *D. discoideum* gene homologous to SRF, *srfA*, was characterized previously (Escalante and Sastre, 1998). The generation of knockdown strains showed that *srfA* is required for several stages of development. The developing structure goes through a stage, the slug, that migrates towards the light. *SrfA* knockdown strains present impaired slug migration (Escalante et al., 2001). These strains also show important defects in spore formation (Escalante and Sastre, 1998; Escalante et al., 2004).

The analysis of the *D. discoideum* genome sequence (Eichinger et al., 2005) allowed the identification of a second SRF homologous gene, *srfB*. In this article the pattern of expression of *srfB* and the structure of the gene are described. The possible biological function of the gene has been approached through the generation of a knockout strain. The strain shows deregulation of actin cytoskeleton functions, such as macropinocytosis and cytokinesis, altered patterns of gene expression at starvation, defects in acquisition of aggregation-specific cell–cell adhesion and chemotaxis, due to impaired cAMP signaling, and deranged post-aggregative development.

## Materials and methods

### Cell culture, transformation and development

*D. discoideum* cells were cultured axenically in HL-5 or AX2 medium. Transformation by electroporation was performed as described (Pang et al., 1999). Transformed cells were selected by treatment with blasticidin (Adachi et al., 1994) or neomycin (G418). Filter development was induced by spreading 1–2 × 10^7^ cells on filters (Shaulsky and Loomis, 1993). For development on agar plates, a drop of 1 × 10^6^ cells was spread on non-nutrient agar (Pergolizzi et al., 2002). For submerged development 1–2 × 10^6^ cells were re-suspended in 2 ml of phosphate-based PDF buffer and placed on plastic multi-well plates (Multiwell, 6 well, FALCON, Becton Dickinson Labware, Franklin Lakes, NJ, USA). For development in shaking suspension, cells were harvested in 0.017 mM Soerensen Na/K phosphate buffer, pH 6.0, resuspended at 1 × 10^7^ cell/ml in Erlenmeyer flasks and shaken at 150 rpm and 23 °C on a rotary shaker (Clim-O-Shaker, A. Kuehner, Birsfelden, Switzerland). Cell treatment with cAMP pulses was done by addition of 2 × 10^− 8^ M cAMP every 6 min, starting within 1 h from beginning of starvation, using a Braun perfusor (Bozzaro et al., 1987).

### Generation of knockout, over-expressing and reporter strains

Flanking regions of the *srfB* gene, including nucleotides − 954 to 80 and 303–1498, in relation to the start codon were generated by PCR and cloned at both sides of the blasticidin resistance gene in the pGEMT-Easy plasmid vector (Promega). For expression, the coding region of the *srfB* gene, from amino acids 2 to 467, was amplified by PCR and cloned in the pCGFP-CTAP vector (kindly provided by Dr. Pauline Schaap). In a subsequent step the Act15 promoter was substituted by a 2-kb long *srfB* promoter fragment (nucleotides − 2152 to 51) generated by PCR.

Putative promoter regions of the *srfB* gene were amplified by PCR and cloned in the PsA-ialphagal vector (Detterbeck et al., 1994), in substitution of the PsA promoter. During amplification of promoter region 1 the last three nucleotides of the 5′UTR, AAG, were changed to ATG to introduce an initiation codon in frame with the reporter gene. β-Galactosidase activity was detected as previously described (Escalante and Sastre, 2006).

### Pinocytosis

For each time point, 2.5 × 10^6^ cells were incubated with 2 mg/ml of FITC-dextran (Sigma-Aldrich, St. Louis, MO, USA). Cells were washed, lysed and the incorporated dextran determined in a fluorimeter, as previously described (Rivero and Maniak, 2006). Experiments were made in duplicate and at least three independent experiments were carried out for each strain.

### F-actin and nuclear staining

The determination of F-actin was made using the F-actin Visualization kit (Cytoskeleton, Inc., Denver, CO, USA), according to the manufacturer's instructions. After F-actin staining, cells were incubated with 0.1 μg/ml of 4,6-Diamidino-2-phenylindole (DAPI), washed and mounted for microscopic observation (Zeiss Axiophot microscope with Plan-Neofluar objectives).

### Northern blot analyses

RNA was isolated using Trizol reagent (Gibco-BRL). RNA transfer to nylon membranes, probe labeling and hybridization were carried out as previously described (Sambrook et al., 1989). DNA probes were generated by PCR using oligonucleotides designed from nucleotide sequences obtained at dicty Base (http://www.dictybase.org).

### Rapid amplification of cDNA ends

RNA was isolated from AX4 cells at proliferation or after 8 h of multi-cellular development. The SMART™ RACE cDNA Amplification kit from Clontech (Clontech Laboratories, Inc., Mountain View, CA, USA) was used for amplification of the 5′ and 3′ untranslated regions of the *srfB* mRNA. The oligonucleotides 5′-CCCTCTTCGCCTGGTGATGATGGAGTTGG-3′, complementary to the region coding for amino acids 20 to 27 of the protein, and 5′-CCACTTCCTTCTATACCATCACC-3′, coding for amino acids 406 to 414, were used. Amplification products were cloned in the pGEMT-Easy vector and the insert from 10 different colonies was sequenced for each of them.

### Microarray hybridization

RNA was isolated from AX4 structures after 8 h of multi-cellular development and from *srfB*^*−*^ structures after 6 h of development. 25 μg of each of three independent RNA samples, from mutant and wild type, were labeled separately with Cy3 and Cy5 by direct incorporation of the dye-dCTP conjugate (GEHealtcare) in a reverse transcription reaction (Superscript III, Invitrogen Carlsbad, CA, USA). The six pairs of complementary labeled cDNAs (three replicates in both dye orientations) were co-hybridized to DNA microarrays comprising 9300 probes printed in duplicate. Arrays were scanned using an Axon Instruments Genepix 4000B scanner and the resulting images quantified (Genepix 3.0, Axon Instruments) and analyzed using the Bioconductor package Limma (Gentleman et al., 2004; Smyth, 2004, 2005). Background fluorescence was subtracted (Kooperberg et al., 2002), and the resulting log ratios normalized by the print-tip loess function of Limma. The overall log ratio for each gene was obtained using linear model fitting (least squares) and the significance of the apparent differential expression was assessed by a Bayesian approach (Smyth, 2004), adjusting the *p*-values to control the false discovery rate (Benjamini and Hochberg, 1995). In the course of the analysis, a set of genes contiguous on chromosome 2 was found, with a mean log ration shifted to approximately − 1, indicating a novel segmental duplication present in the control cells used. These genes were omitted from the list of differentially expressed genes.

### Cell adhesion assay

At the end of growth, AX4 and *srfB*-null cells were washed in Soerensen phosphate buffer pH 6.0, resuspended at 1 × 10^7^ per ml and incubated under shaking. At the starvation times indicated cells were washed, resuspended at a final concentration of 1 × 10^7^ per ml and incubated in Soerensen phosphate buffer, with or without 10 mM EDTA, in 0.2 ml volume cuvettes. Cell adhesion was measured by using the light scattering assay and the agglutinometer of Beug and Gerisch, as described (Bozzaro et al., 1987; Bozzaro, 2006).

### Chemotaxis assay

Cells starved in shaken culture for 4 to 9 h were plated onto 35-mm diameter glass-based dishes (Iwaki) at a density of approximately 1 × 10^5^ cell/cm^2^. Chemotaxis was tested by local stimulation with a microcapillary (Femtotips 1, Eppendorf), filled with a 1.0 mM cAMP solution. Cells were observed with a 20× or a Neofluar 100×/1.3 oil immersion objective, equipped with DIC filter. Images were captured with intervals varying between 0.66 and 1.8 s and recorded in a Panasonic video-recorder (AG-TL7007) with ZVS-47DE camera (Zeiss) mounted on a Zeiss Axiovert HAL200 microscope (Arigoni et al., 2005; Pergolizzi et al., 2002). The recorded time-lapse movies were analyzed using the Image J software. Cell tracks for at least 20 cells in each field (for a total of 170 cells for AX4 and 100 for *srfB*^*−*^ cells) were analyzed and cell speed calculated by measuring the distance over time. Orientation was determined by measuring the angle between two subsequent positions of each cell in relation to the position of the cAMP-delivering capillary. Movies showing the migration of AX4 and *srfB*^*−*^ cells are included as Supplementary material.

## Results

**Fig. 1:**
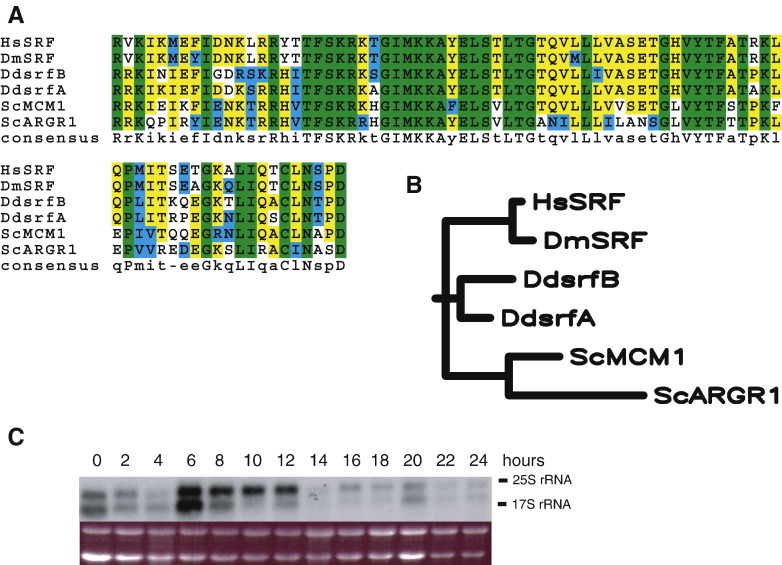
Identification and expression of the *srfB* gene. Panel A shows the comparison of the deduced amino acid sequences of the *srfB* encoded protein (DdsrfB) with those of *D. discoideum srfA* gene (DdsrfA), human (HsSRF) and *D. melanogaster* (DmSRF) SRF proteins and *S. cerevisiae* Mcm1 (ScMCM1) and ArgR1 (ScARGR1) proteins. Amino acids that are identical in all the proteins are boxed in green and those conserved in 5 of the proteins in yellow. Sequence alignments were made using the ClustalW program at the San Diego Supercomputer Centre (http://www.workbench.sdsc.edu). A phylogenetic tree made from these alignments using the neighbor-joining method, with a generator seed of 111 and 1000 bootstrap trials, is shown in panel B. Panel C, *srfB* expression in proliferating cells (time 0) and at different times of multi-cellular development (2 to 24 h), as determined by Northern blot (upper panel). Migration of ribosomal RNAs is indicated at the right. Ethidium bromide staining of the gel is shown in the lower panel.

Analysis of the *D. discoideum* genome detected the existence of a second gene coding for a protein similar to MADS-box transcription factors of the SRF family, in addition to the previously characterized *srfA* gene (Escalante and Sastre, 1998). This gene, *srfB*, codes for a protein of 467 amino acids. The predicted amino acid sequence of a region of the protein (amino acids 38 to 118) showed high similarity to the MADS-box and SAM (SRF, Agamous, MCM1) domains of SrfA from *D. discoideum* (86.4% identity), SRF from *Homo sapiens* (72.8%) and *Drosophila melanogaster* (72.8%) and the *Saccharomyces cerevisiae* homologues MCM1 (71.6%) and ARGR1 (56.8%) (Fig. 1A). A phylogenetic tree derived from these sequences also shows that SrfB is more closely related to SrfA than to the animal or yeast genes, indicating a possible duplication of the *srf* gene in Dictyostelids after their divergence from animals and fungi (Fig. 1B).

*SrfB* expression in proliferating cells and during multicellular development was studied by Northern blot (Fig. 1C). Maximal expression was observed during growth and between 6 and 12 h of development. Two mRNAs were detected that are differentially regulated since the larger mRNA is the predominant species between 8 and 12 h of development.

**Fig. 2:**
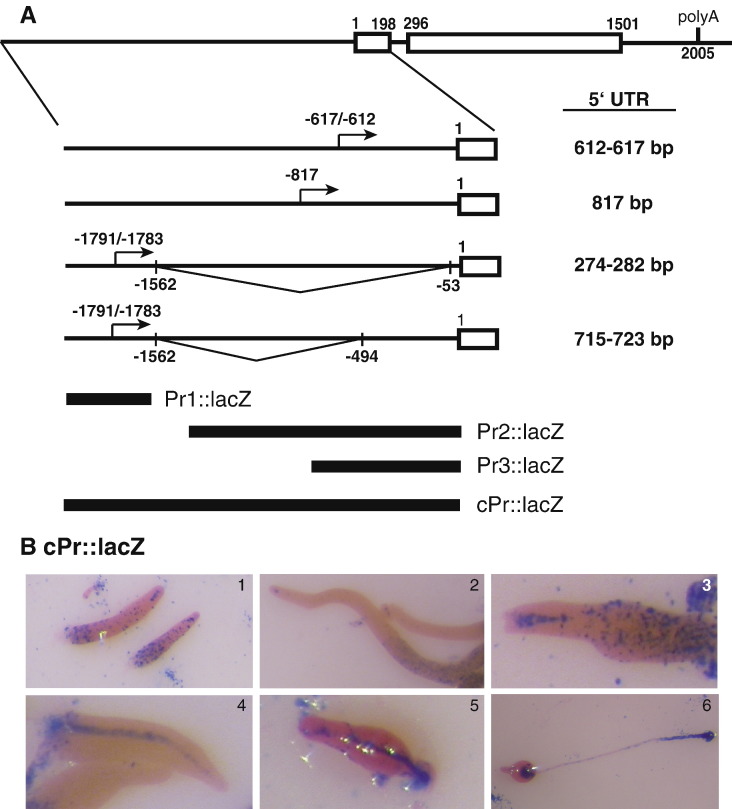
Structure and cell-type specific function of the *srfB* promoter region. The nucleotide sequence of the *srfB* mRNA 5′ and 3′ untranslated regions was determined by rapid amplification of the cDNA ends (RACE) and the results are schematically shown in panel A. Exon-encoded Open Reading Frames are indicated as boxes and introns and untranslated sequences as lines. The single polyadenylation site identified is indicated (PolyA). The three different transcription initiation regions are indicated by arrows. Two alternative splicing patterns of the mRNAs transcribed from the more distal promoter are indicated. The position of transcription initiation sites, splicing donor and acceptor sites, intron/exon boundaries and polyadenylation site are indicated in relation to the translation initiation codon. The size of the different 5′ untranslated regions of the mRNAs is indicated at the right. The promoter fragments fused to the lacZ reporter gene for expression analyses are schematically indicated (Pr1∷lacZ, Pr2∷lacZ and Pr3∷lacZ). The intergenic region, up to the closest upstream gene, was considered the complete promoter region (cPr∷lacZ). Panel B shows the detection of β-galactosidase activity, indicative of *lacZ* expression, under the control of the complete *srfB* promoter region. The following developmental stages are shown: first finger (1), slug (2), finger (3), early culminant (4, 5) and fruiting body (6).

The origin of the different mRNAs was investigated by the amplification of both cDNA ends. Amplification of the 5′ untranslated region of the cDNA (5′UTR) showed the existence of four different mRNAs coding for the same protein (Fig. 2A). Two of the mRNAs were transcribed from transcription initiation sites located between nucleotides − 1783 and − 1791 in relation to the ATG initiation codon. Transcripts initiated at these sites were spliced from a donor splicing site located at nucleotide − 1562 to two different splice-acceptor sites, located at nucleotides − 53 and − 494. In addition, two transcription initiation regions, located at − 817 and between − 612 and − 617 were detected. These transcripts were not spliced in their 5′ UTRs. The analysis of the 3′ UTR detected a single polyadenylation site 504 nt downstream of the protein stop codon, that is preceded by a consensus polyadenylation addition signal.

To determine the spatial and temporal pattern of transcriptional activity, each promoter region (Pr1–3) and the complete promoter region (cPr) were cloned in reporter vectors containing a modified *lacZ* gene coding for unstable β-galactosidase, as schematically shown in Fig. 2A. The intergenic region upstream of the *srfB*-coding region, 4 kb long, was considered as the complete promoter region in these studies. The region of promoter 1 under study contained a 934 nt long fragment upstream of initiation sites − 1791/− 1783 and also included the mRNA 5′ UTR to the splicing donor site. The Pr2 construct included a 1218 nt region, starting downstream of the splicing donor site up to the initiation codon. The Pr3 construct included a 786 nt fragment from just downstream of the − 817 transcription initiation site to the initiation codon.

The complete promoter (cPr) directed *lacZ* expression in vegetative growing cells. Expression decreased during mound formation to be later induced in some cells dispersed throughout the structure (not shown). In later stages of development expression was specifically observed in prestalk cells located at the PstAB region of the first-finger structures, as well as in cells dispersed in the posterior region (Fig. 2B1). Expression was almost completely lost in migratory slugs, except for a few prestalk cells located at the pstAB region (Fig. 2B2). Later on, *lacZ* expression was observed in prestalk cells that were migrating from the top of the structure towards the substrate to form the stalk and in the lower cup region (Fig. 2B3–6). No *lacZ* expression was detected at the prespore region or in the sorus (Fig. 2B5–6).

**Fig. 3:**
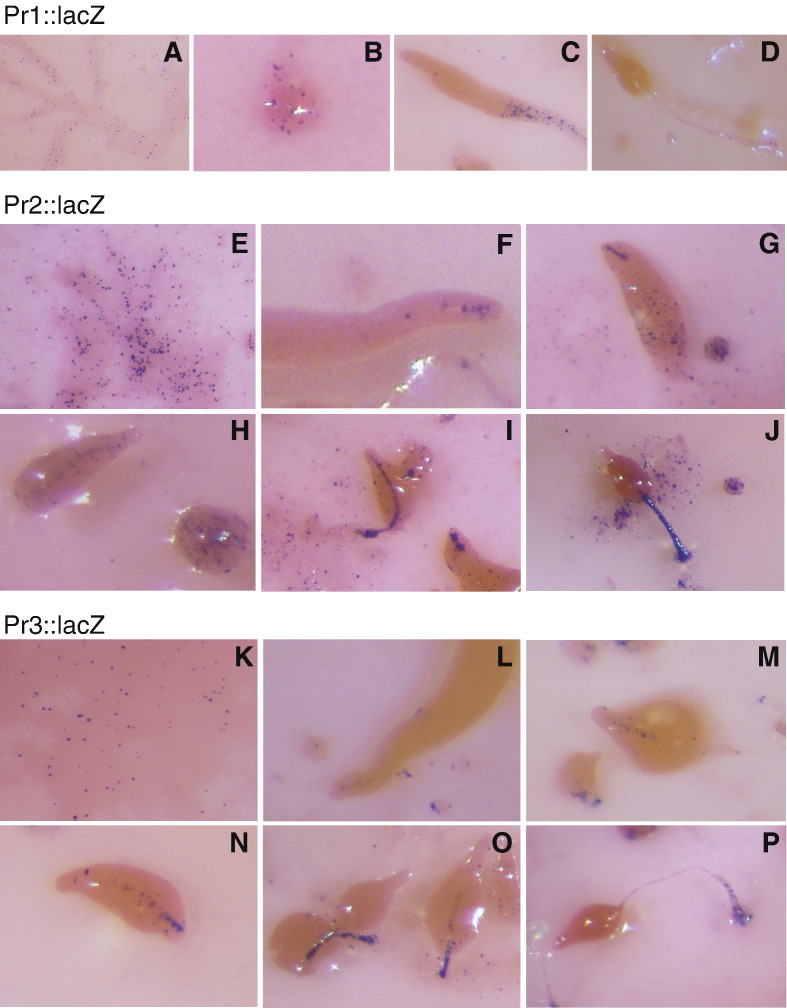
Developmental regulation of *srfB* promoters. Expression of the *lacZ* reporter gene under the control of *srfB* promoter 1 (A–D), 2 (E–J) or 3 (K–P) was determined by X-gal staining. Aggregating cells (A, E, K), mounds (B), slugs (C, F, L), fingers (G, M), early culminant (H, I, N, O) and culminant structures (D, J, P) were analyzed.

Promoter 1 directed *lacZ* expression in growing cells, in cells dispersed through the streams (Figs. 3A, B) and in some cells located at the posterior end in early structures (Fig. 3C), but no expression was observed in fruiting bodies (Fig. 3D). In contrast, promoter 2 almost entirely reproduced the pattern of expression of the complete promoter. Therefore, *lacZ* expression directed by promoter 2 was observed in growing cells, in dispersed cells in the streams (Fig. 3E) and in the posterior region of intermediate structures (Fig. 3G), in the PstAB prestalk region of first finger and slug structures (Figs. 3F, G), and in the stalk of early and late culminant structures (Figs. 3H–J). However, the level of *lacZ* expression detected with the Pr2 construct was significantly weaker than that obtained with the complete promoter. Promoter 3 induced a pattern of *lacZ* expression, similar to that of promoter 2, in streams (Fig. 3K), prestalk (Figs. 3L–N) and stalk regions (Figs. 3O, P) but its activity was much weaker.

### Generation of a *srfB* knockout strain

**Fig. 4:**
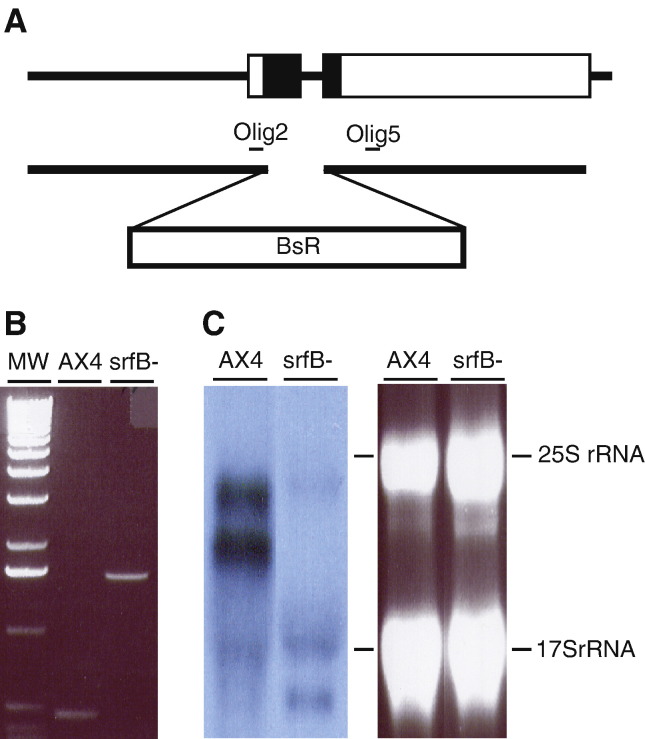
Generation of a mutant strain where the *srfB* gene has been interrupted. The plasmid construct used for *srfB* interruption and partial deletion is schematically shown in panel A. The *srfB* coding region is indicated as a box in the upper diagram, with the MADS-box coding region shown as a black box. The lower diagram indicates the two genomic regions amplified by PCR and cloned at both sides of the blasticidin-resistance cassette (BsR). The position of the oligonucleotides used for verification of the mutation (Olig2 and Olig5) is indicated. Panel B shows the results of PCR reactions primed with oligonucleotides Olig2 and Olig5 using genomic DNA obtained from wild type (AX4) or *srfB* mutant (*srfB*^*−*^) cells. Migration of the 1 kb Molecular Weight Marker (Invitrogen) is indicated (MW). Panel C, the expression of *srfB* mRNA in wild type (AX4) and *srfB* mutant cells (*srfB*^*−*^) was analyzed by Northern blot (left panel). The right panel shows the ethidium bromide staining of the gel. Migration of the ribosomal RNAs is indicated at the right.

A mutant strain where the *srfB* gene is interrupted was generated to study its function. In this strain, part of the MADS-box coding region of *srfB* (amino acids 27 to 69) was substituted with the blasticidin resistance gene (Fig. 4A). One colony was identified in which the *srfB* gene was interrupted, as shown by PCR analyses (Fig. 4B). Cells from this strain did not express *srfB* mRNAs (Fig. 4C). Mutant cells grew slightly more slowly than wild type (WT) cells on bacteria and in shaken culture with axenic media (data not shown).

**Fig. 5:**
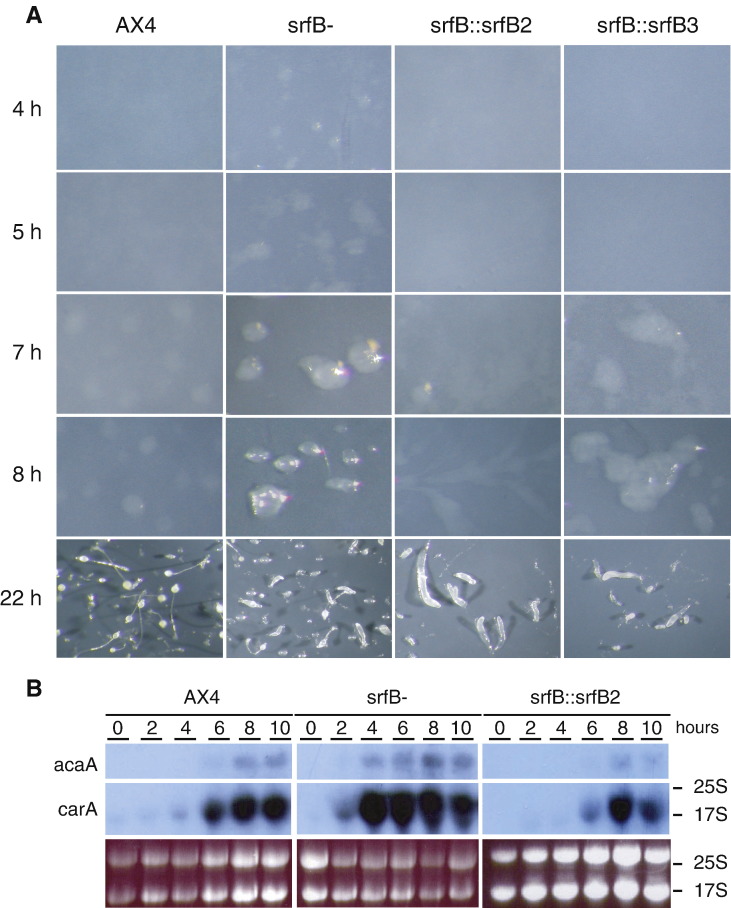
Early multicellular development of the *srfB* mutant strain. Panel A: Cells from wild type (AX4), *srfB* mutant (*srfB*^*−*^) strains and *srfB* mutant strains transfected with a plasmid for *srfB* expression (*srfB*∷*srfB2* and *srfB*∷*srfB3*) were plated on nitrocellulose filters to induce multicellular development. Pictures were taken at 4, 5, 7, 8 and 22 h of development. Panel B: RNAs obtained from wild type (AX4), *srfB* mutant (*srfB*^*−*^) and *srfB* mutant strains transfected with a plasmid for *srfB* expression (*srfB*∷*srfB2*) proliferating cells (0) or cells developed on nitrocellulose filters for 2, 4, 6, 8 or 10 h were analyzed for expression of the genes coding for cAMP receptor A (*carA*) or the adenylate cyclase A (*acaA*). Migration of the ribosomal RNAs is indicated for the *carA* blot since the *acaA* mRNA migrated more slowly than both rRNAs. The ethidium bromide staining of the gel is shown in the lower panel.

Mutant cells were able to form cell aggregates on nitrocellulose filters or non-nutrient agar about 2 h earlier than wild type (AX4) cells (Fig. 5A). The expression of some early genes required for aggregation, such as *carA* (coding for the cAMP receptor A) or *acaA* (coding for adenylyl cyclase A) was also induced 2 h earlier in mutant than in WT strains (Fig. 5B).

To determine if the alteration observed was due to the interruption of the *srfB* gene, a plasmid containing the *srfB* gene under the transcriptional control of its own promoter was transfected into the *srfB*^*−*^ cells. Two different clones that expressed *srfB* (*srfB*∷*srfB*2 and *srfB*∷*srfB*3) showed a temporal pattern of aggregation on nitrocellulose filters similar to the one of WT cells (Fig. 5A). Mutant cells expressing *srfB* showed a pattern of *carA* and *acaA* expression similar to that of wild type cells, as shown for the srfB∷srfB2 strain (Fig. 5B).

**Fig. 6:**
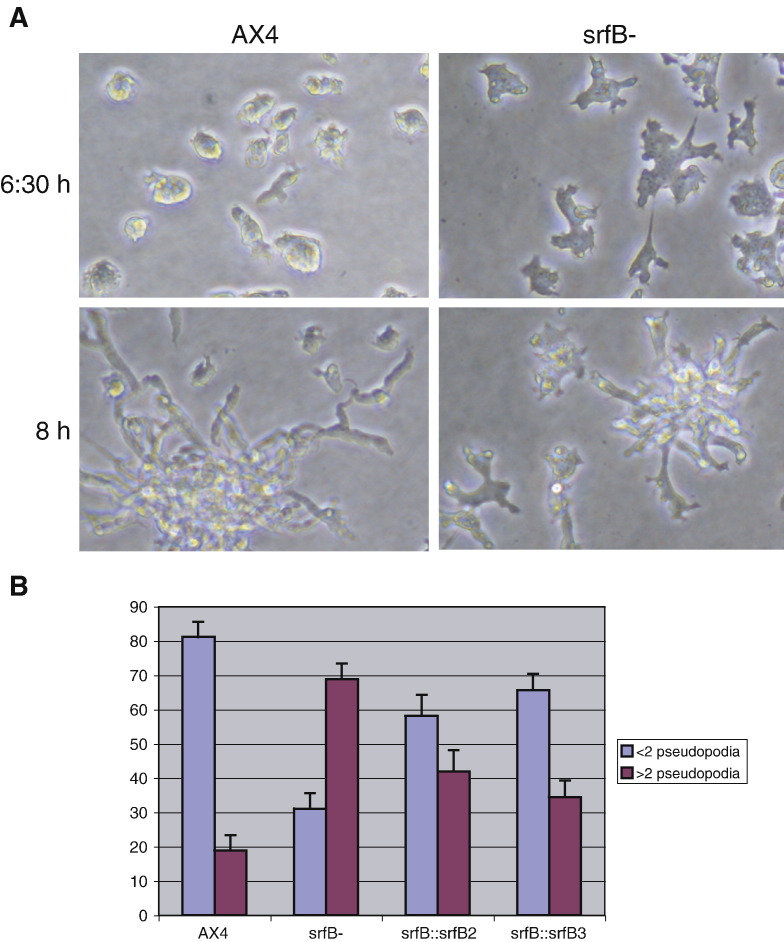
Aggregation of wild type and *srfB* mutant cells. Panel A: Wild type (AX4) or *srfB* mutant (*srfB*^*−*^) cells were incubated on plastic plates for 6:30 or 8 h. Pictures of early stages of cell activation (6:30 h) and cell streaming (8 h) are shown. Panel B: The morphology of wild type (AX4) or *srfB* mutant (*srfB*^*−*^) cells, and mutated cells that express *srfB* (*srfB*∷*srfB2* and *srfB*∷*srfB3*) was analyzed. Cells that presented 2 or more pseudopodia were quantified in three different experiments. The mean and standard deviation, represented as percentage of analyzed cells, are indicated.

Observation of cell aggregation under submerged conditions detected significant differences between mutant and WT cells. During aggregation WT cells polarized and organized into streams, where the leading edge of one cell contacts the rear end of the preceding one (Fig. 6A). In contrast, *srfB*^*−*^ cells extended pseudopodia in different directions during aggregation and hardly formed streams (Fig. 6B). The number of pseudopodia formed by the *srfB*^*−*^ strain was determined and compared to that of WT cells. The mutant strain formed significantly more pseudopodia than WT cells (Fig. 6B). Mutant strains expressing *srfB* partially reverted the defect observed, and formed less pseudopodia than the mutant strain (Fig. 6B). When incubated on a glass slide, aggregation-competent WT cells formed very large streams. In contrast, mutant cells formed small clumps from the beginning of starvation, their size increased by accretion and large streams were never formed (data not shown).

**Fig. 7:**
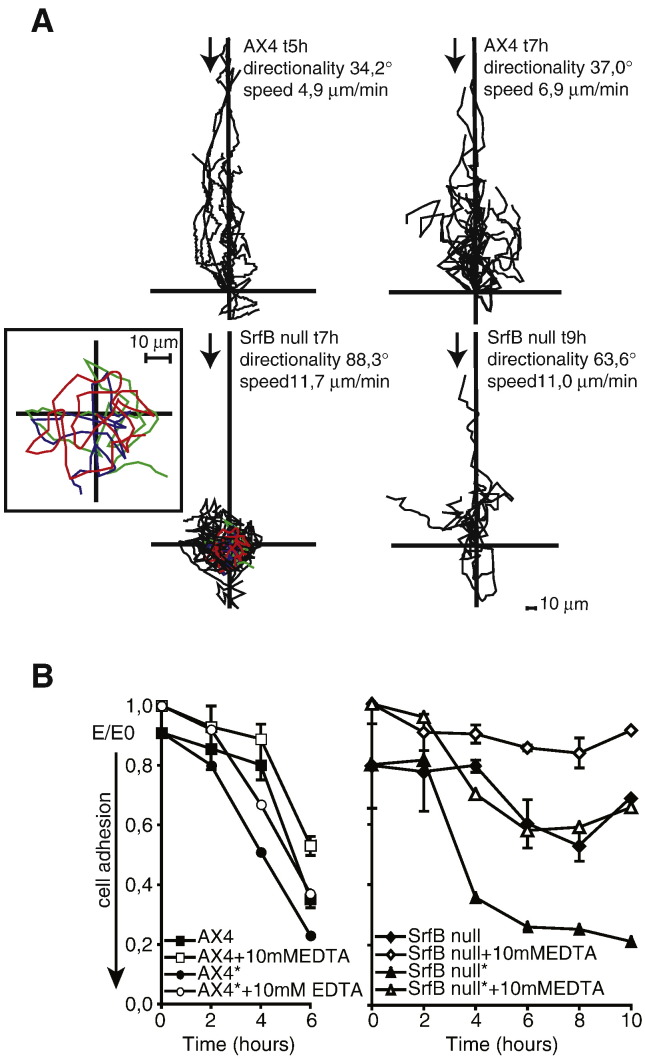
Chemotaxis and cell adhesion of wild type and *srfB* mutant cells. Panel A: Wild type (AX4) or *srfB* mutant (*srfB*^*−*^) cells were starved under shaking for the time indicated, washed and plated on glass-based dishes. The movement of the cells towards cAMP, diffusing from a micropipette, was recorded and analyzed using ImageJ software. Migration speed and directionality of cells was measured as described in Materials and methods. Arrows indicate the source of the cAMP gradient. The insert is an enlargement of three representative tracings (in colors), to better show cell movement around, or close to, the cell axis. The distance in μm from the origin is also indicated. Panel B: Determination of EDTA-sensitive and EDTA-stable adhesion for AX-4 and *srfB*^*−*^ cells. AX4 and *srfB*-null cells were incubated under shaking. At the times indicated in the abscissa, AX4 (on the left) and *srfB*^*−*^ (on the right) cells were washed, resuspended at a final concentration of 1 × 10^7^ per ml and incubated with (open symbols) or without (closed symbols) 10 mM EDTA in 0.2 ml volume cuvettes. Samples containing control and mutant cells treated with pulses of 20 nM cAMP every 6 min, starting at time 0 are marked with asterisks. Aggregation was measured by using the light scattering assay as described in Materials and methods. The light scattering assay measures unscattered light (*E* = extinction) at equilibrium, i.e. after 40 to 60 min of incubation. Unscattered light is high when cells are single and diminishes with increasing cell clumping. The values are normalized for *E*_0_ (the value of EDTA-treated, totally dissociated cells at time 0). A value of 1 corresponds to single cells and lower *E*/*E*_0_ values correlate with formation of larger and more compact aggregates. Mean values, with error bars, of two separate experiments in duplicate are shown.

The functional significance of the morphological defect observed was further studied by analyzing chemotaxis to cAMP. Starving cells were plated on glass-based dishes, stimulated with cAMP diffusing from a microcapillary and the migration of cells towards cAMP was analyzed. In contrast to WT cells, *srfB*^*−*^ cells showed impaired migration after either 7 or 9 h of starvation (movies of the cells are included as Supplementary material). Mutant cells displayed high spontaneous motility in comparison to WT cells but directionality and migration towards cAMP were very deficient (Fig. 7A). *srfB*^*−*^ cells stuck strongly to the substrate at the rear end, and moved mainly around their axis, especially after 7 h of starvation. Orientation towards cAMP of the mutant cells improved after 9 h of starvation but is still significantly impaired with respect to WT cells (Fig. 7A).

The finding that *srfB*^*−*^ cells, starved in shaken cultures, failed to aggregate normally prompted us to assay their ability to form EDTA-stable contacts. EDTA-stable cell adhesion is developmentally regulated and strictly dependent on the expression of the contact site A glycoprotein on the cell surface (Harloff et al., 1989). The encoding *csA* gene belongs to a battery of genes required for aggregation, which are induced at very low level upon starvation and whose expression is strongly stimulated by endogenous cAMP pulses (Gerisch, 1987). Thus, measuring adhesion in the presence or absence of EDTA is a rapid and established method to assess acquisition of aggregation competence (Bozzaro, 2006; Gerisch, 1987).

At the beginning of starvation mutant cells displayed increased EDTA-labile adhesion compared to AX4 cells, which induced rapid formation of small clumps (Fig. 7B). In contrast to the parental strain, which formed EDTA-stable cell–cell contacts by 6 h of development, the mutant cells displayed only a very slight increase in EDTA-stable adhesion even after 10 h of starvation, suggesting strong inhibition in the acquisition of developmentally regulated cell–cell adhesion (Fig. 7B).

To assess whether the defect was due to impaired cAMP signaling, cells were treated with cAMP pulses, supplied every 6 min, thus mimicking the endogenous periodic release of cAMP. As shown in Fig. 7B, cAMP pulsing rescued the mutant, resulting in appearance of EDTA-stable contacts by 6–8 h of development. As expected, acquisition of EDTA-stable adhesion was accelerated by cAMP pulses in the parental AX4 cells (Fig. 7B). Thus, these results exclude possible functional defects in contact sites A-mediated cell adhesion or in the ability of the mutant to respond to cAMP pulses. They suggest, instead, that the *srfB*-null cells are impaired in the periodic release of cAMP, which is essential for *csA* gene expression.

In contrast to cell adhesion, the chemotaxis defect was rescued only in part by cAMP pulses. Cells polarized and oriented more rapidly toward the capillary, but the strong cell attachment to the substrate at the rear end persisted for most cells and the rate of chemotactic motility toward the capillary was thus only slightly increased (*data not shown*). More important, the chemotaxing cells failed to form streams; they moved toward the cAMP source either as single cells or as small flat clumps, like cAMP-unpulsed cells. Taken together, these results strongly suggest that *srfB*-null cells are impaired in cAMP signal relay, not however in cAMP sensing.

Despite these problems in chemotaxis and cell adhesion, *srfB*^*−*^ cells formed aggregates on nitrocellulose filters or agar 2 h earlier than wild type cells, as mentioned above. No streams were observed and aggregation resulted from rapid formation of small clumps that grew larger, possibly by accretion with other small clumps.

**Fig. 8:**
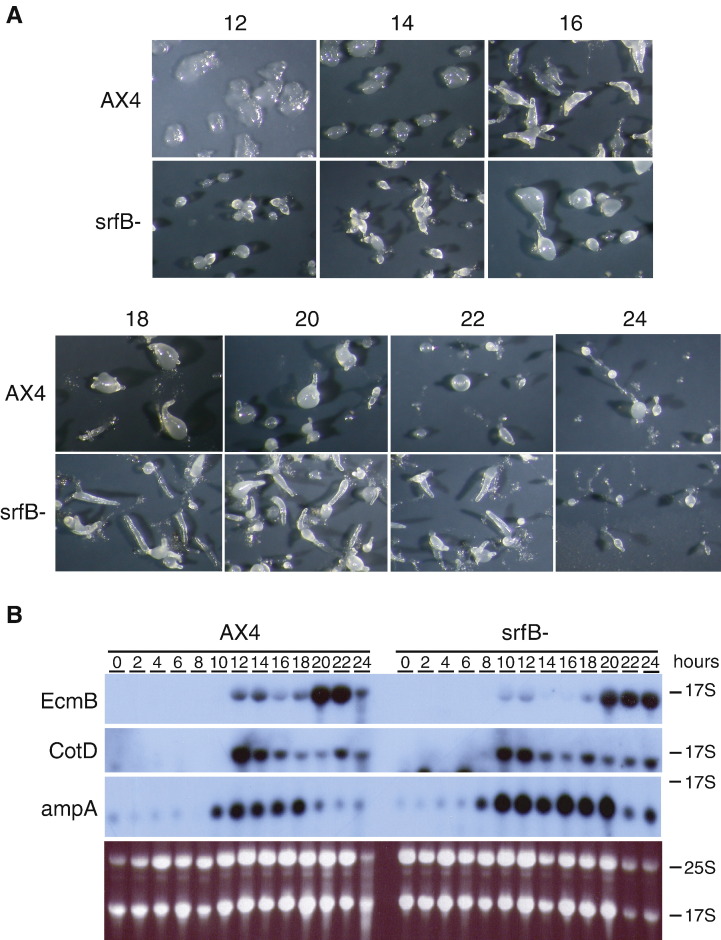
Post-aggregation development of wild type and *srfB* mutant strains. Panel A: Wild type (AX4) or *srfB* mutant (*srfB*^−^) cells were deposited on nitrocellulose filters to induce multicellular development. The structures found at middle and late stages of development (12, 14, 16, 18, 20, 22 and 24 h) are shown. Panel B: RNA was obtained from wild type (AX4) and *srfB* mutant (*srfB*^−^) cells during proliferation (0) or at different stages of development on nitrocellulose filters (2–24 h). The three upper panels show the hybridization with a probe for the prestalk-specific gene *ecmB*, the prespore-specific gene *cotD* and the *ampA* gene. Ethidium bromide staining of a representative gel is shown in the lower panel. Migration of the ribosomal RNAs is indicated at the right.

Post-aggregative multicellular development was completed by the *srfB* mutant strain and fruiting bodies were formed 24 h after starvation (Fig. 8A). However, the mutant strain showed an increased tendency to form migratory slugs under these conditions, that favor direct culmination in WT strains (18–22 h of development in Fig. 8A). Slug formation was also observed when cells were grown on bacteria and resulted in the presence of numerous trails left on the plates by migrating slugs (data not shown). Developmental timing was also analyzed by the expression of the prestalk-specific *ecmB* and the prespore-specific *cotD* genes. The expression of both genes was induced 2 h earlier in the *srfB*^*−*^ strain (Fig. 8B). However, *ecmB* induction at culmination occurred at the same time (20 h) in *srfB*^*−*^ and WT strains. A similar change in the pattern of expression was observed for the *ampA* gene, which was induced 2 h earlier in the mutant and maintained high-expression level for a longer time than the wild type strain (Fig. 8B). Spores formed by the *srfB* mutant strain were more heterogeneous in size and shape than WT spores but no difference in viability was observed.

### Actin cytoskeleton-related functions in growing cells

**Fig. 9:**
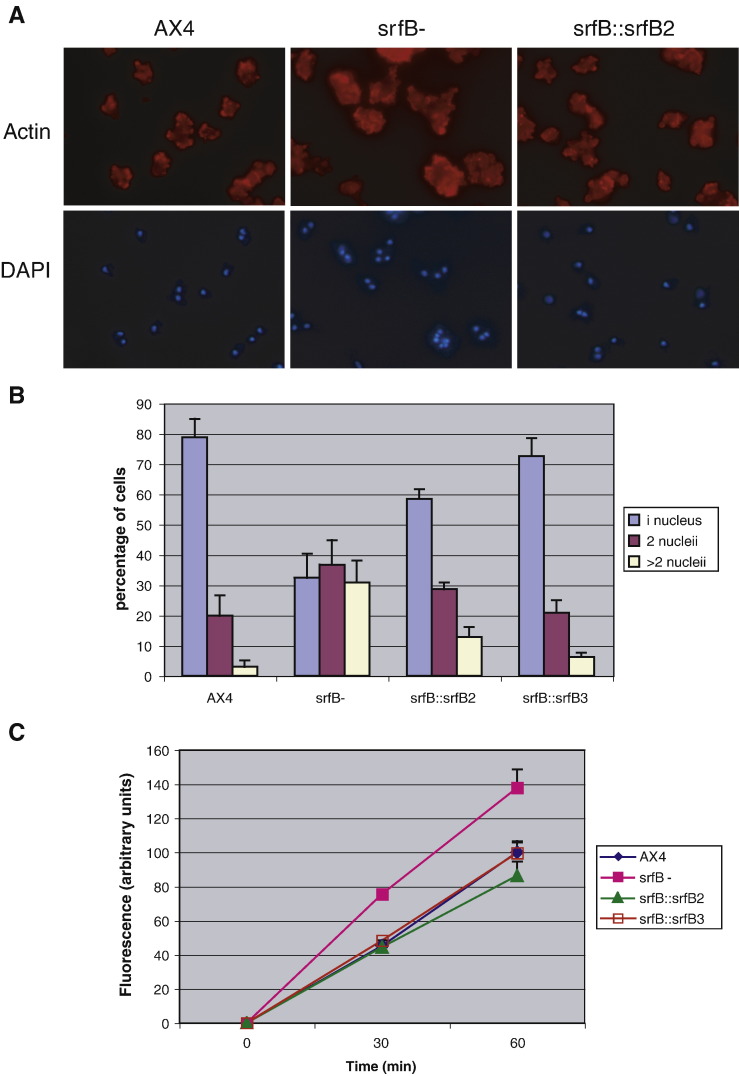
Study of cytokinesis and macropinocytosis in wild type and *srfB* mutant cells. Panel A: Wild type cells (AX4), *srfB* mutant (*srfB*^−^) cells or mutant cells that express *srfB* (*srfB*∷*srfB*2) were grown on plastic plates and stained for F-actin (actin) and with the DNA staining reagent 4,6-diamidino-2-phenylindole (DAPI). Panel B: Quantification of the percentage of cells that present 1, 2 or more than 2 nuclei in wild type (AX4) *srfB* mutant (*srfB*^−^) or mutant cells that express *srfB* (*srfB*∷*srfB*2, *srfB*∷*srfB*3). Mean and standard deviations are represented. Panel C: Pinocytosis was measured by the uptake of FITC-dextran present in the culture media. Mean and standard deviations of wild type (AX4), *srfB* mutant (*srfB*^*−*^) cells and mutant cells that express *srfB* (*srfB*∷*srfB*2, *srfB*∷*srfB*3) at 30 and 60 min of incubation are represented.

*SrfB*^*−*^ cells are larger and more heterogeneous in size than WT cells when growing on plastic surfaces. The mutant strain presented a significantly higher proportion of cells with two or more nuclei, as compared with WT cells (Fig. 9A). Mutant cells over-expressing *srfB* partially reverted the larger proportion of multi-nucleated cells (Fig. 9B).

Since impaired actin cytoskeleton function might produce defects in cytokinesis, which could result in larger cells, another actin cytoskeleton-dependent process, macropinocytosis, was investigated. Incorporation of media containing FITC-dextran was faster by *srfB* mutant cells than by WT cells (Fig. 9C). The difference in ingestion rate was of 1.6 times. *SrfB*^*−*^ cells that over-express *srfB* showed a macropinocytosis rate similar to the one of WT cells (Fig. 9C).

### Analyses of *srfB*-dependent gene expression

DNA microarrays containing 9300 probes (for approximately 8600 different genes) were hybridized to identify genes whose expression was dependent on *srfB*. RNAs were obtained from WT and *srfB*^*−*^ strains at early mound stage. A large number of genes are differently expressed in the *srfB*^*−*^ and WT strains. Using a cutoff of *p* < 0.001, a total of 276 were over-expressed in the *srfB* mutant strain and 301 were under-expressed. Among them, 53 genes were over-expressed and 157 under-expressed more than 2 fold in the mutant strain. Since only one time point was used, the microarray data do not allow one to distinguish whether the differences observed between strains are due to altered timing or altered expression level, and therefore they have to be considered preliminary. For this reason, gene expression was analyzed by Northern blot for some representative genes.

**Fig. 10:**
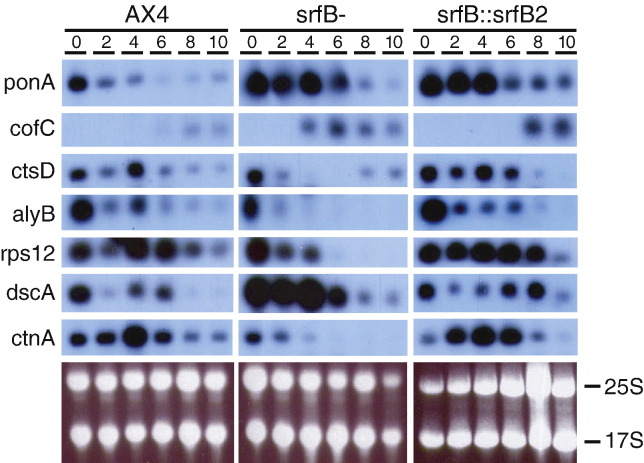
Developmental expression of *srfB*-regulated genes. RNA was obtained from wild type (AX4), *srfB* mutant (*srfB*^−^) cells and mutant cells expressing *srfB* (*srfB*∷*srfB*2) during proliferation (0) or at different developmental stages (2–10). The expression of the genes coding for actin cytoskeleton regulatory proteins (Ponticulin (*ponA*), cofilin 2 (*cofC*)), lysosomal proteins (preprocathepsin D (*ctsD*), lysozyme (*alyB*)), ribosomal proteins (ribosomal protein S12 (*rps12*)), and genes involved in multicellular development (Discoidin (*dscB*), countin (*ctnA*)) is shown. Ethidium bromide staining of representative gels is shown in the lower panel. Migration of the ribosomal RNAs is indicated to the right of each panel. The mRNAs corresponding to the genes analyzed in this figure migrated faster than the 17S ribosomal RNA.

The genes coding for ponticulin (*ponA*) and cofilin 2 (*cofC*), involved in actin cytoskeleton structure and regulation, are over-expressed more than 2 fold in the mutant. Northern blot analyses showed that the ponticulin-coding gene, *ponA*, was expressed during proliferation and repressed after starvation. The expression of this gene was maintained for a longer time in the *srfB*^*−*^ strain (Fig. 10). Similarly, the gene coding for cofilin 2 (*cofC*) was induced earlier and to higher levels in the mutant cells (Fig. 10). Mutant cells expressing *srfB* showed a pattern of *ponA* and *cofC* expression more similar to that of wild type cells than to the mutant ones (*srfB*∷*srfB*2, Fig. 10).

There are 14 genes coding for lysosomal proteins and 24 genes coding for ribosomal proteins that are expressed at levels at least 2 fold lower in the mutant than in the WT strain. For example, genes coding for lysozyme (*alyA, B*, *C*) and preprocathepsin D (*ctsD*) are some of the more importantly down-regulated genes in the mutant (7 and 10 fold, respectively). Northern blot analyses indicated that lysosomal (*ctsD*, *alyB*) and ribosomal (*rps12*) protein encoding genes are repressed at starvation. Repression is faster and more pronounced in the *srfB*^*−*^ strain, in concordance with the lower expression detected with the microarrays (Fig. 10). SrfB expression in mutant cells induced a pattern of *alyB*, *ctsD* and *rps12* expression similar to that of wild type cells.

Some of the differentially expressed genes are regulated at the transition between growth and development, or have a role in development or cell differentiation. Among them, discoidin genes (A, B and C chains) are up-regulated, and two countin genes (*ctnA*, *ctnC*) are down-regulated in the *srfB* mutant. Again these putatively differentially expressed genes were analyzed by Northern blot. The discoidin coding gene *dscA* showed higher expression in *srfB*^*−*^ than in WT cells (Fig. 10). The countin coding gene, *ctnA*, was expressed at lower levels in *srfB* mutant cells and repressed at earlier developmental stages than in WT cells (Fig. 10). Expression of *srfB* in mutant cells resulted in an increase in *ctnA* expression and a decrease in *dscA* expression after starvation, making expression patterns similar to the wild type ones.

## Discussion

Analysis of the *D. discoideum* genome detected the existence of two SRF-homologous genes, *srfA*, previously characterized, and *srfB*, that is the subject of this study. The animal species whose genome sequence is known, have a single SRF homologous gene, while *S. cerevisiae* contains two (*mcm1* and *argR1*) and plants possess numerous genes (Theiben et al., 1996). Phylogenetic analyses indicate that *D. discoideum* and *S. cerevisiae* SRF-homologous genes were originated by gene duplication after the divergence of animal, fungi and Dictyostelids.

*SrfB* is expressed during proliferation and multicellular development under the control of different promoter regions. The distal promoter directs transcription in vegetative cells and in cells scattered in the mound and finger structures. The two more proximal promoters direct expression in vegetative cells, cells scattered at the mound stage and prestalk (PstAB region) and stalk cells in developing structures. However, some regulatory regions controlling the activity of these promoters must be dispersed in the intergenic region because reporter vectors that contain this complete region recapitulate the pattern of expression of the three promoters, directing higher expression levels than any of them. It is remarkable that the 5′UTR regions (up to 817 bp) and the introns present in the 5′UTR (1066 and 1509 bp long) are unusually large in comparison to other *D. discoideum* genes, that present a mean intron size of 146 bp (Eichinger et al., 2005).

The *srfB* homologous gene, *srfA*, is also transcribed from several promoters that direct expression to different regions of the structure, although expression patterns are very different for both genes (Escalante et al., 2001).

The biological functions in which *srfB* is involved have been studied by the generation of a mutant strain, where part of the MADS-box coding region of the gene has been substituted by a blasticidin-resistance gene. *SrfB* mutant cells showed several defects both during growth and development, consistent with the complexity of the promoter.

During proliferation *srfB*^*−*^ cells showed alterations in cytokinesis and pinocytosis. They also showed impaired migration and adhesion of the rear end to the substrate during early development. These defects could be indicative of impaired actin cytoskeleton function.

Mutant cells also displayed an altered starvation response, in comparison to WT cells. *SrfB* mutant cells seem to respond to starvation faster than WT cells. Aggregates are formed on nitrocellulose or agar about 2 h earlier in *srfB*^*−*^ than in WT cells. Some early aggregation genes, such as those coding for the cAMP receptor (*carA*) or adenylyl cyclase A (*acaA*) are also induced about 2 h earlier in the mutant. In addition, some of the genes that are quickly repressed after starvation, such as those coding for ribosomal proteins (Ken and Singleton, 1994; Agarwal et al., 1999) and lysosomal enzymes (Bush et al., 1994) are down-regulated in the mutant. Discoidin is also induced earlier and to higher levels in the mutant. The developmental timing of expression of these genes is reverted when srfB is expressed in the mutant strain. Complex changes in gene expression have been described for *D. discoideum* cells upon starvation (Iranfar et al., 2001). The data obtained for the mutant indicate that *srfB* could be involved in these changes at the proliferation/development transition.

In apparent contrast to these observations, *srfB* mutant cells showed a developmental delay when incubated in shaken cultures, because chemotaxis to cAMP, the main process that directs cell aggregation, and acquisition of EDTA-stable cell adhesion are highly impaired. This apparent contradiction can be solved by assuming that early aggregate formation on nitrocellulose filters or agar is mainly due to clumping of cells by random collision. In favor of this interpretation is the increased EDTA-labile adhesion measured in the mutant that may explain rapid formation of small clumps, in the absence of chemotaxis. Stream formation, which is typical of chemotaxing cells, is not observed on agar. Aggregate formation could make possible subsequent steps of development on nitrocellulose filters or agar.

Chemotaxis studies with cAMP diffusing from a microcapillary show that mutant cells are impaired in migration towards cAMP. Despite their increased motility and a reduced though clear orientation ability, the *srfB*^*−*^ cells do not become polarized and do not migrate or migrate poorly towards the cAMP source. Instead, the cells move around their axis, remaining mainly attached with their rear end to the glass substrate.

Developmentally regulated EDTA-stable cell adhesion is also strongly delayed under shaking. EDTA-stable adhesion is dispensable for aggregation on nitrocellulose filter or agar, though contributing to optimal aggregation and becoming essential under more stringent cell–substrate interactions (Harloff et al., 1989; Ponte et al., 1998). Nevertheless, EDTA-stable adhesion is a good marker of cAMP-dependent aggregation-specific gene expression (Gerisch, 1987).

Remarkably, cAMP pulsing restores EDTA-stable adhesion, indicating that the *srfB*^*−*^ null mutant is able to respond to cAMP, but is defective in generating endogenous cAMP signals, which are necessary for expression of the csA glycoprotein and other genes required for aggregation (Mehdy and Firtel, 1985; Gerisch, 1987; Iranfar et al., 2003). This conclusion is further supported by the finding that cAMP-pulsed cells fail to form streams, when stimulated by cAMP diffusing from a microcapillary, indicating a defect in cAMP signal relay. Although polarization and cell orientation toward the cAMP source were strongly improved by cAMP pulses, a third defect of the mutant, namely the strong attachment of the cell uropod to the substratum or to small clumps was rescued only minimally, resulting in a modest increase in the chemotaxis index.

Understanding the molecular basis of such a complex phenotype requires additional experiments that will be done in the future. It is important to find out if the defect in cAMP signaling is upstream or downstream of the heterotrimeric G protein and whether misregulation is at the level of adenylyl cyclase or cAMP phosphodiesterase activity or in any other component of the cAMP signalling. A protein involved in cAMP wave propagation, the phophodiesterase inhibitor PDI, is for example overexpressed 2.5 fold in the mutant (data not shown).

Correlating the defect with *srfB*-gene activity ultimately requires identification of the cis-acting target sites at DNA level. A few conclusions can be, however, drawn from these experiments: all phenotypic defects of the mutant during aggregation, with exception of the unusually strong cell adhesion to the substrate, can be explained with defective cAMP signaling. Although we cannot exclude that SrfB may directly regulate expression of one or more genes involved in cAMP signaling, it is more likely that the defect in cAMP signaling is secondary to misregulation of genes involved in the transition from growth to development. As shown by the gene expression data, several genes involved in this transition are indeed either down- or over-expressed in the mutant.

We have no explanation for the localized strong adhesion of the cells at their rear end to the glass substrate, and to our knowledge this is the first time that such a phenotype has been described. Overexpression of ponticulin and cofilin 2 could in part explain this phenotype. Ponticulin is an integral plasma membrane protein that binds actin filaments to the membrane. Cells lacking ponticulin aggregate sooner than WT cells (Hitt et al., 1994). Cofilin 2 stimulates turn-over of actin filaments. This protein is located at the cell–substrate adhesion sites (Aizawa et al., 2001). Over-expression of the related cofilin-1 gene produces enhancement of spontaneous cell motility (Aizawa et al., 1996), as also observed in the srfB mutant. Over-expression of these proteins could explain cell spreading and increased adhesion to the substrate, but not the localized cell attachment at the uropod.

In addition, two genes coding for countin are strongly down-regulated in the mutant (ctnA-5, 6 fold; ctnC-5, 4 fold and Fig. 10). Countin participates in the determination of the size of the structures by regulating cell–cell adhesion (Roisin-Bouffay et al., 2000). Countin mutant cells also show decreased F-actin level, and increased cell motility and cell–cell adhesion (Tang et al., 2002). Therefore, the decrease in ctnA and ctnC expression could also contribute to some of the defects observed in cell motility and cell–cell adhesion in the *srfB* mutant.

*D. discoideum* development continues from aggregation through two alternative pathways. In the presence of ammonia the structures lay on their sides to originate a migratory structure (slug) (Hashimoto and Matsui, 1988). If ammonia does not accumulate, the fruiting body is directly formed at the site of aggregation (Kristen et al., 2005). The mechanisms that regulate slug formation versus culmination are not well understood but the cells at the tip of the structure seem to have a regulatory role in this process (Chisholm and Firtel, 2004). *SrfB* mutant structures show a marked tendency to form slugs under conditions that favor direct culmination of WT structures. The expression of *srfB* in the prestalk PstAB cells, and in the first cells that migrate towards the substrate to form the stalk, might indicate that *srfB* could participate in the process of initiation of culmination.

SRF regulates the expression of many components of the actin cytoskeleton and contractile apparatus in animals, including actin and myosin isoforms (Miano et al., 2007). However, no changes in the expression of actin or most of myosin isoforms have been detected in the microarray analyses of the *srfB* mutant. Only an actin-related protein and, at lower levels, myoI and the essential and regulatory myosin light chains show changes of expression in the mutant (data not shown). Regulation of actin and myosin expression is, however, complex in *D. discoideum* due to the existence of 30 genes coding for actin (Eichinger et al., 2005), 13 for myosin heavy chain and 4–6 for myosin light chains (Kollmar, 2006). Analyses of the nucleotide sequence detected the existence of possible SRF-binding sites in the putative promoter region of the *actin15*, Myosin A and *mlcR* genes. The regulatory function of these putative SRF binding sites is presently under investigation. However, no consensus SRF-binding site (CCA/T_6_GG) was identified in the analyses of the putative promoter region of the genes whose expression is altered in the *srfB*^*−*^ strain during development. This observation further supports the hypothesis mentioned above for the cAMP-dependent gene expression, namely that the regulation by SrfB could be indirect, through the regulation of the expression of other transcription factors at the transition from growth to development. The analyses of the promoter region of 24 SrfA-dependent genes did not show the presence of any consensus SRF-binding site either. Because of this reason, the possibility that the SRF-binding site could have changed should be considered and a functional analysis of these promoters will be required to ascertain their transcriptional regulation by SrfB.

Globally considered, the data obtained indicate that *srfB* plays important roles in the regulation of the proliferation/development transition and in the initiation of culmination during fruiting body formation. Future studies, directed at identifying the regulated target genes, may help in furnishing a mechanistic interpretation of *srfB* gene activity.
